# Advanced Glycation End Products Induce Endothelial-to-Mesenchymal Transition via Downregulating Sirt 1 and Upregulating TGF-*β* in Human Endothelial Cells

**DOI:** 10.1155/2015/684242

**Published:** 2015-02-01

**Authors:** Wei He, Jian Zhang, Tian-yi Gan, Guo-jun Xu, Bao-peng Tang

**Affiliations:** Pacing Electrophysiology Division, First Affiliated Hospital of Xinjiang Medical University, No. 137, Liyushan South Road, Urumqi, Xinjiang 830054, China

## Abstract

In the present study, we examined the advanced glycation end products- (AGEs-) induced endothelial-to-mesenchymal transition (EndMT) in human umbilical vein endothelial cells (HUVECs). Results demonstrated that AGE-BSAs significantly reduced the cluster of differentiation 31 (CD 31) expression, whereas they promoted the expression of fibroblast-specific protein-1 (FSP-1), *α*-smooth muscle antibody (*α*-SMA), and collagen I at both mRNA and protein levels in HUVECs. And the AGE-BSAs also promoted the receptors for AGEs (RAGEs) and receptor I for TGF-*β* (TGFR I) markedly with a dose dependence, whereas the Sirt 1 was significantly downregulated by the AGE-BSA at both mRNA and protein levels. Moreover, the Sirt 1 activity manipulation with its activator, resveratrol (RSV), or its inhibitor, EX527, markedly inhibited or ameliorated the AGE-mediated TGF-*β* upregulation. And the manipulated Sirt 1 activity positively regulated the AGE-induced CD31, whereas it negatively regulated the AGE-induced FSP-1. Thus, Sirt 1 was confirmed to regulate the AGE-induced EndMT via TGF-*β*. In summary, we found that AGE-BSA induced EndMT in HUVECs via upregulating TGF-*β* and downregulating Sirt 1, which also negatively regulated TGF-*β* in the cell. This study implied the EndMT probably as an important mechanism of AGE-induced cardiovascular injury.

## 1. Introduction

Risk for the development of atherosclerosis is enhanced in diabetes mellitus (DM), which leads to an increased risk for such cardiovascular complications as stroke, myocardial infarction, and even death [1, 2]. And numerous reports suggest that systemic metabolic abnormalities in diabetes mellitus, such as hyperglycemia, hyperinsulinemia, and dyslipidemia, are associated with accelerated atherosclerosis [3–5]. However, exact mechanisms responsible for the acceleration of atherosclerosis remain elusive. Advanced glycation end products (AGEs), which develop mainly via the Maillard reaction [6], accumulate in various tissues at an extremely accelerated rate in diabetes mellitus [7–9]. It has been confirmed that AGEs are implicated in the pathogenesis of diabetic microvascular and macrovascular complications [10–13]. AGEs have been reported to stimulate several signaling pathways. Increased AGEs promote intracellular reactive oxygen species (ROS) and nitric oxide (NO), as well as the mitogen-activated protein kinase (MAPK) cascade, which, through intermediate molecules, activates different targets including transcription factors such as nuclear factor kappa-light-chain-enhancer of activated B cells (NF-*κ*B) and activator protein 1 (AP-1) [14–16]. And the activated downstream signals at least in part promote the apoptosis [17–19].

Such epithelial cells as renal tubular epithelial cells and retinal pigment epithelial cells have phenotypic changes and thus no longer resemble the normal cell populations from which they originated [20, 21]. This transdifferentiation is a hallmark of epithelial-to-mesenchymal transition (EMT), by which epithelial cells lose their epithelial phenotypes and acquire mesenchymal, fibroblast-like properties, show reduced intercellular adhesion, and show increased motility [22, 23]. Moreover, AGEs have been also indicated to stimulate tube formation and migration of vascular endothelial cells dose dependently [24, 25]. This implies that AGEs can elicit angiogenesis and contribute to the development and progression of diabetic microangiopathy [26]. This may account for the disabilities and high mortality rates in patients with this disease [27]. Recently, studies have found that, as a special part of epithelial cells, endothelial cells also can transdifferentiate into mesenchymal cells in a similar way, which is called endothelial-to-mesenchymal transition (EndMT), physiologically or physiopathologically [28–31]. Particularly, a more recent study reported that AGEs induced EndMT in cultured endothelial cells [32]. Therefore, the EndMT promotion by AGEs and the detailed mechanism underlining it might shed light on the cardiovascular injury by AGEs.

EndMT is a complex biological process in which endothelial cells lose their specific endothelial cell markers, such as cluster of differentiation 31 (CD 31) and E-cadherin, and acquire a mesenchymal or myofibroblastic phenotype initiating expression of mesenchymal cell products including fibroblast-specific protein-1 (FSP-1), *α*-smooth muscle actin (*α*-SMA), and type I interstitial collagens [33]. In contrast to the very extensive studies conducted to unravel the molecular mechanisms and the regulatory pathways implicated in EMT [34, 35], the mechanisms involved in the EndMT process have rarely been explored. Limited studies have unraveled that TGF-*β*/Smad and TGF-*β*/Snail signaling play crucial roles in the induction of EndMT [36–41], via employing a TGF-*β* receptor kinase inhibitor which inhibits the activation of TGF-*β* [42] as well as several small molecule inhibitors of intracellular phosphorylation reactions [38, 40]. Besides the TGF-*β*, silent mating type information regulation 2 homolog 1 (Sirt 1) has also been shown to be implicated in the AGEs-induced EndMT, via inhibiting the expressions of fibronectin and TGF-*β*1 in glomerular mesangial cells [43]. The downregulated Sirt 1 might be also involved in the regulation of AGE-induced EndMT in human umbilical vein endothelial cells (HUVECs).

In the present study, we investigate the EndMT induction by AGEs in HUVECs via examining the expression of endothelial cell marker, CD 31, and mesenchymal cell markers, FSP-1, *α*-SMA, and collagen I. And we also examined the induction of both TGF-*β* and Sirt 1 in the AGE-treated HUVECs. This study implied important regulatory roles by TGF-*β* and Sirt 1 in the AGE-induced EndMT of HUVECs.

## 2. Materials and Methods

### 2.1. Cell Culture, Treatment, and Reagents

Human umbilical vein endothelial cells (HUVECs) were purchased from American Type Culture Collection (ATCC, Rockville, MD, USA) and were maintained in Kaighn's modification of Ham's F-12 medium (F-12 K medium, Invitrogen, Carlsbad, CA, USA) containing 10% fetal calf serum (FBS, Gibco, Rockville, MD, USA), supplemented with 100 U/L penicillin and 10 mg/L streptomycin (Invitrogen, Carlsbad, CA, USA). Cells were incubated in a humidified atmosphere containing 5% CO_2_ at 37°C and propagated every 5 days at a split ratio of 1 : 4 using trypsin (Ameresco, Framingham, MA, USA). For assessment of the effect of AGE-BSA on endothelial cells, approximately 85% confluent HUVEC cells were incubated with F-12 K medium containing 2% FBS and 25, 50, 100, or 300 *μ*g/mL AGE-BSA or BSA for 48 h or for 96 h, and then they were collected for the mRNA or protein analysis or for Sirt 1 activity analysis. For the experiments to investigate the regulation on AGE-BSA-mediated EndMT by Sirt 1 regulator, cells were supplemented with the Sirt 1 activator, resveratrol (RSV) with 2 or 10 *μ*M (Sigma-Aldrich, St. Louis, MO, USA), or the Sirt 1 inhibitor, Ex527 with 0.1 or 0.5 *μ*M (Santa Cruz Biotechnology, Santa Cruz, CA, USA).

### 2.2. Preparation and Characterization of AGE-BSA

AGE-BSA was prepared using D-glucose (Sigma-Aldrich, St. Louis, MO, USA) and bovine serum albumin (BSA, Thermo Scientific, Rockford, IL, USA) as previously described [44, 45]. In brief, 50 mg/mL BSA was incubated with (Glu-BSA) or without (Control) 0.25 M D-glucose in 0.2 M phosphate buffered saline (PBS) (pH 7.4) at 37°C for 8 weeks in dark, using 50 mg/mL BSA prepared by the same incubation without D-glucose as control. All preparations of AGEs and BSA control were dialyzed in 10 mM of PBS (pH 7.4) for 96 h to remove the free glucose and passed over Detoxi-Gel Columns (Detoxi-Gel Endotoxin Gel, Thermo Fisher Scientific, Rockford, IL, USA) to remove endotoxin. The protein concentration was determined by BCA assay (Thermo Fisher Scientific, Rockford, IL, USA). And the glycation of AGE-BSA was examined by spectrofluorometry (PerkinElmer, Waltham, MA, USA) with excitation wavelength of 370 nm and emission wavelength of 440 nm.

### 2.3. RNA Isolation, Reverse Transcription, and qPCR

Total cellular RNA from HUVECs was purified with TRIzol agent (Thermo Scientific, Rockford, IL, USA) according to the manufacturer's manual and was supplemented with RNase inhibitor (Takara, Tokyo, Japan). Real-time quantitative reverse transcription-PCR (RT-qPCR) was performed with Takara One Step RT-PCR kit (Takara, Tokyo, Japan). And the relative quantification was determined using the ΔΔCt method using *β*-actin as reference gene [46]. The primers used were available upon request.

### 2.4. Western Blotting Assay

Protein samples from HUVEC Cell were isolated with the cytoplasm extraction buffer (Thermo Fisher Scientific, Rockford, IL, USA) and quantified by the BCA protein assay kit (Thermo Fisher Scientific, Rockford, IL, USA). Then the protein samples were separated by 12% SDS-PAGE and were transferred to PVDF membranes (Invitrogen, Carlsbad, CA, USA). Target protein bands in the PVDF membranes were probed with rabbit polyclone antibodies to CD 31 (Abcam, Cambridge, UK), FSP-1 (Abcam, Cambridge, UK), *α*-smooth muscle actin (*α*-SMA, LifeSpan BioSciences, Seattle, WA, USA), collagen I, receptor for advanced glycation end product (RAGE, Sigma-Aldrich, St. Louis, MO, USA), TGF-*β* (Sigma-Aldrich, St. Louis, MO, USA), transforming growth factor receptor I (TGFR I, Sinobio, Beijing, China), Sirt 1 (Santa Cruz Biotechnology, Santa Cruz, CA, USA), Sirt 2 (Santa Cruz Biotechnology, Santa Cruz, CA, USA), or *β*-actin (Sinobio, Beijing, China). Goat anti-rabbit IgG (Pierce, Rockford, IL, USA) secondary antibody conjugated to horseradish peroxidase and ECL detection systems (SuperSignal West Femto, Pierce) were used for target protein detection.

### 2.5. Sirt 1 Deacetylase Activity Detection

Fluor de LysSirt1 assay kit (Biomol, Plymouth Meeting, PA, USA) was used to measure the Sirt 1 deacetylase activity, according to the manufacturer's protocol. In brief, HUVECs posttreatment were lysed with 200 *μ*L immunoprecipitation buffer (Abcam, Cambridge, UK). Rabbit antibody to Sirt 1 was incubated at 4°C overnight with precleared lysates, which then were added with 20 *μ*L protein agarose A/G beads (Sinobio, Beijing, China) and were rotated for 2 h at 4°C. The beads were extensively washed and transiently centrifuged before the deacetylation assay. First, the beads were mixed with 35 *μ*L of assay buffer at 37°C for 30 min before the addition of a reaction mixture (15 *μ*L) containing 20 *μ*M fluorosubstrate peptide and 100 *μ*M NAD+ at 37°C for another 30 min; then, the action was stopped by the addition of 50 *μ*L of 1X develop reagent and nicotinamide (2 mM) for 45 min; lastly, the fluorescence was subsequently measured by excitation at 360 nm and emission at 460 nm using a microplate reader (Bio Red, Hercules, CA, USA).

### 2.6. Statistical Evaluations

Statistical analyses were performed using GraphPad Prism software (GraphPad Software, La Jolla, CA, USA). Statistical evaluations are presented as mean ± SE. Data were analyzed using Student's *t*-test. A *P* value < 0.05 or less was considered statistically significant.

## 3. Results

### 3.1. AGE-BSA Induces EndMT in Cultured HUVECs

**Figure 1:**
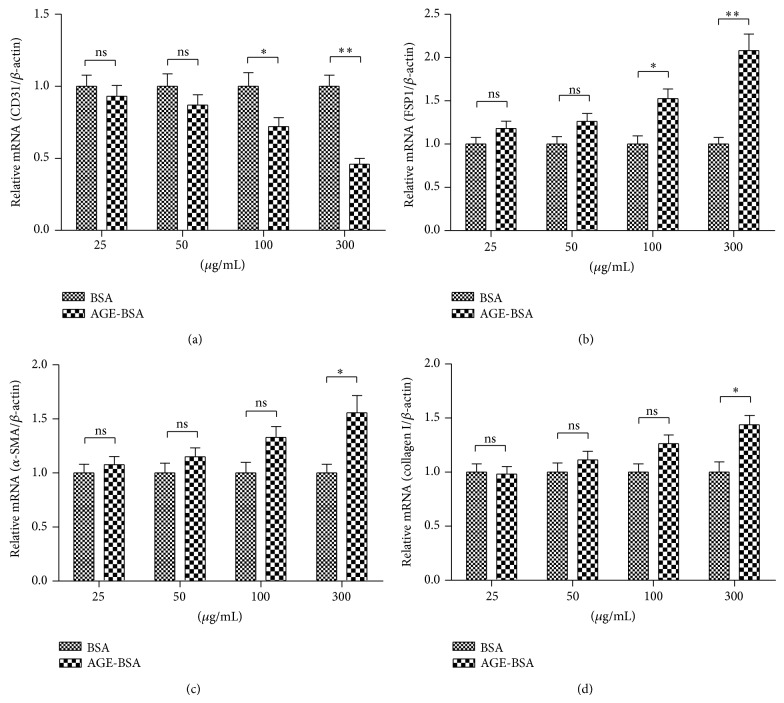
AGE-BSA regulated EndMT-associated molecules in mRNA level. Relative mRNA level of CD 31 (a), FSP-1 (b), *α*-SMA (c), and collagen I (d) to *β*-actin in HUVECs which were treated with 25, 50, 100, or 300 *μ*g/mL AGE-BSA or BSA for 48 h. Each value was averaged for triple independent experiments; statistical significance was shown as ^*^ *P* < 0.05, ^**^ *P* < 0.01; ns: no significance.

To elucidate the AGEs-exerted direct injury to human endothelial cells, we determined the regulation by AGEs in HUVECs on the expression of endothelial cell marker, CD 31, and mesenchymal cell markers, FSP-1, *α*-SMA, and collagen I. HUVECs were exposed to AGE-BSA or BSA (as control) with various concentrations. As shown in Figure 1(a), after 48 h of inoculation, 100 or 300 *μ*g/mL AGE-BSAs significantly reduced the CD 31 mRNA expression in HUVECs, compared to the BSA with the same concentration (*P* < 0.05 or *P* < 0.01). On the other side, such mesenchymal cell markers as FSP-1 (*P* < 0.05 for 100 *μ*g/mL or *P* < 0.01 for 300 *μ*g/mL), *α*-SMA (*P* < 0.05 for 300 *μ*g/mL), and collagen I (*P* < 0.05 for 300 *μ*g/mL) were significantly upregulated by the AGE-BSAs treatment, rather than the BSA treatment.

**Figure 2:**
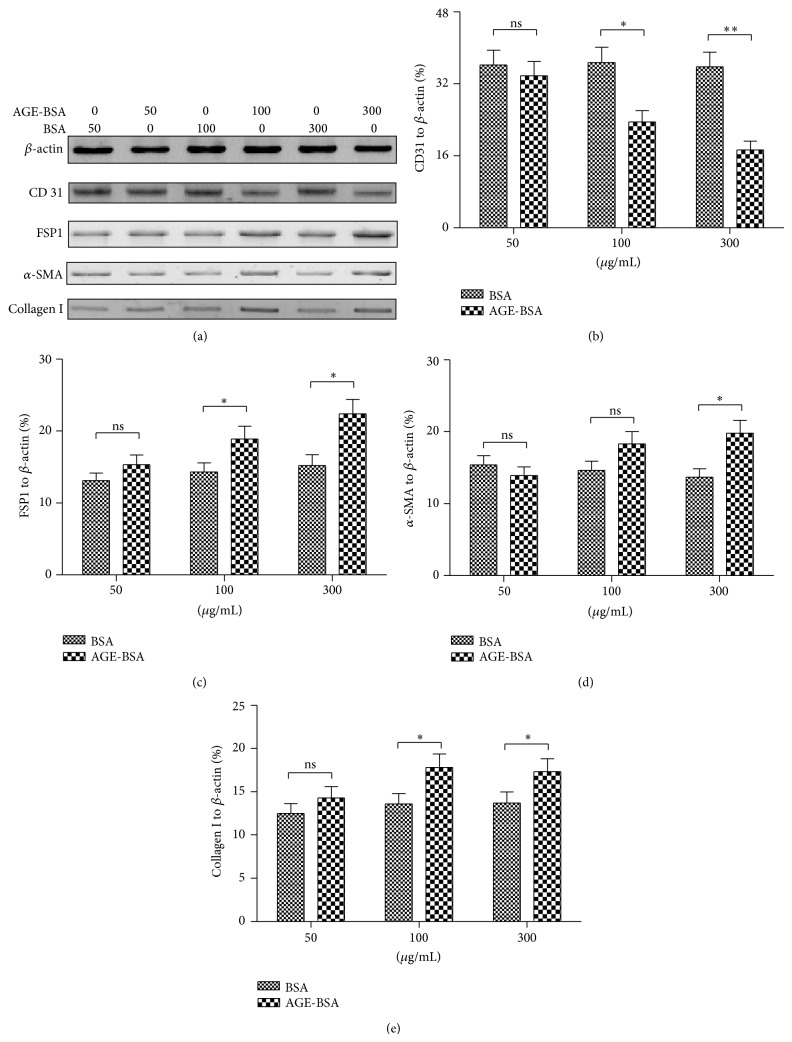
EndMT-associated molecules were regulated by AGE-BSA in protein level. (a) Western blot assay of CD 31, FSP-1, *α*-SMA, collagen I, and *β*-actin in HUVECs which were treated with 50, 100, or 300 *μ*g/mL AGE-BSA or BSA for 96 h. ((b)–(e)) Relative level of CD 31 (b), FSP-1 (c), *α*-SMA (d), and collagen I (e) to *β*-actin in HUVECs which were treated with AGE-BSA or BSA with various concentrations. Each value was averaged for triple independent experiments; statistical significance was shown as ^*^ *P* < 0.05, ^**^ *P* < 0.01; ns: no significance.

To further confirm the transition of endothelial characteristic to mesenchymal characteristic, that is, EndMT, which was induced by AGEs, we then examined, in protein level by western blot assay, the endothelial or the mesenchymal markers in the HUVECs, subject to AGE-BSA or BSA for 96 h. It was demonstrated that CD 31 was also significantly downregulated by the AGE-BSA treatment with 100 or 300 *μ*g/mL (Figures 2(a) and 2(b); *P* < 0.05 or *P* < 0.01). Whereas the protein levels of mesenchymal markers were significantly upregulated by the AGE-BSA treatment (Figures 2(a), 2(c), 2(d), and 2(e)), the upregulation in protein level developed in the 100 *μ*g/mL group (*P* < 0.05 for either FSP-1 or collagen I) and in the 300 *μ*g/mL group (*P* < 0.05 for either FSP-1, *α*-SMA, or collagen I). These observations suggest that AGEs induce EndMT in endothelial cells.

### 3.2. AGE-BSA Treatment Promotes the TGF-*β*1 Expression in HUVECs

**Figure 3:**
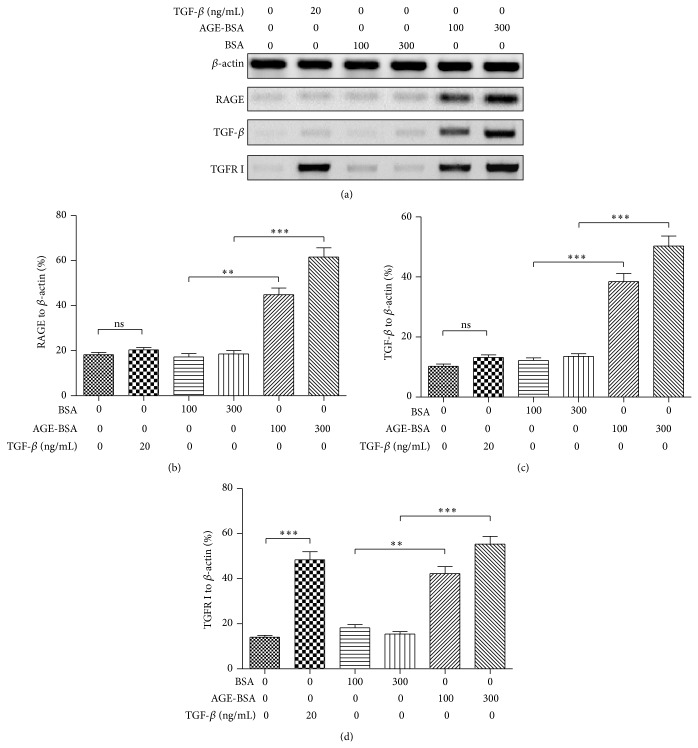
AGE-BSA upregulated TGF-*β* in HUVECs. (a) Western blot assay of RAGE, TGF-*β*, TGFR I, and *β*-actin in HUVECs which were treated with 100 or 300 *μ*g/mL AGE-BSA or BSA for 96 h; in another group cells were treated with 20 ng/mL TGF-*β*. ((b)–(d)) Relative level of RAGE (b), TGF-*β* (c), and TGFR I (d) to *β*-actin in HUVECs. Each value was averaged for triple independent experiments; statistical significance was shown as ^**^ *P* < 0.01, ^***^ *P* < 0.001; ns: no significance.

EndMT shares lots of signaling pathways with epithelial-to-mesenchymal transition (EMT) and can be induced* in vitro* by transforming growth factor beta 1 (TGF-*β*1) [47, 48]. In order to investigate the molecular signaling pathway in the EndMT induced by AGEs, we then examined the TGF-*β*1 activation in the HUVECs after AGE treatment. Western blot analysis of the AGE-treated HUVECs demonstrated that AGEs promoted the expression of the receptor for advanced glycation end product (RAGE), which is usually promoted by and bound to AGEs [49, 50]. And it was indicated that RAGE was significantly promoted by 100 or 300 *μ*g/mL AGE-BSA treatment, in contrast to the BSA treatment (Figures 3(a) and 3(b); *P* < 0.01 or *P* < 0.001). Moreover, as shown in Figure 3(c), the TGF-*β*1 in protein level was also markedly promoted by the 100 or 300 *μ*g/mL AGE-BSA treatment (*P* < 0.001). To examine the regulation by AGEs on the receptor for TGF-*β*1, we firstly confirmed the TGFR I promotion by TGF-*β*1 treatment (20 ng/mL for 48 h; *P* < 0.001) in HUVECs (Figure 3(d)). Then we examined the TGFR I level in the AGE-BSA- or BSA-treated HUVECs with 100 or 300 *μ*g/mL, and a significantly high level of TGFR I was also confirmed in the AGE-BSA-treated cells (*P* < 0.01 for 100 *μ*g/mL or *P* < 0.001 for 300 *μ*g/mL). Taken together, the AGE-BSA promoted the activity of TGF-*β* in the human endothelial cells.

### 3.3. AGE-BSA Treatment Decreases the Expression and the Activity of Sirt 1 in HUVECs

**Figure 4:**
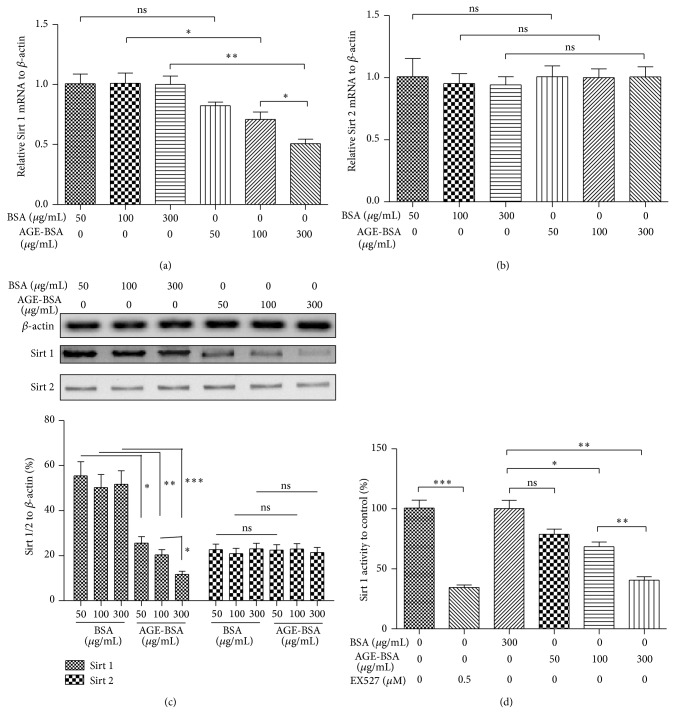
Sirt 1 was promoted by AGE-BSA in HUVECs. ((a) and (b)) Relative mRNA level of Sirt 1 (a) or Sirt 2 (b) to *β*-actin in HUVECs which were treated with 50, 100, or 300 *μ*g/mL AGE-BSA or BSA for 48 h. (c) Western blot analysis of Sirt 1 and Sirt 2 in protein level in AGE-BSA- or BSA-treated HUVECs. (d) Relative Sirt 1 activity in HUVECs which were treated with 50, 100, and 300 *μ*g/mL AGE-BSA or 300 *μ*g/mL BSA for 96 h. All experiments were performed in triplicate, and statistical significance was considered when *P* < 0.05 or less, ^*^ *P* < 0.05, ^**^ *P* < 0.01, and ^***^ *P* < 0.001; ns: no significance.

Sirt 1 has recently been reported to decrease in diabetes [51, 52] and is far more recently confirmed to resist AGE-induced, TGF-*β*1-dependent EMT in glomerular mesangial cells [43]. To identify the possible regulatory role of Sirt 1 in the AGE-induced EndMT in endothelial cells, we analyzed the expression and the activity of Sirt 1 in AGE-treated HUVECs. Expression of both Sirt 1 and Sirt 2 was examined in BSA- or AGE-BSA-treated HUVECs. It was shown in Figure 4(a) that Sirt 1 mRNA was significantly downregulated by the AGE-BSA (*P* < 0.05 for 100 *μ*g/mL or *P* < 0.01 for 300 *μ*g/mL), dose dependently (*P* < 0.05 for 100 *μ*g/mL versus 300 *μ*g/mL). However, the Sirt 2 mRNA was not significantly regulated by either AGE-BSA or BSA (Figure 4(b)). And the western blot analysis of the two molecules also indicated a significantly downregulated Sirt 1 in protein level in the AGE-BSA-treated cells (Figure 4(c); *P* < 0.05 for 50 *μ*g/mL, *P* < 0.01 for 100 *μ*g/mL, or *P* < 0.001 for 300 *μ*g/mL), with a dose dependence (*P* < 0.05 for 100 versus 300 *μ*g/mL), whereas the Sirt 2 was not significantly regulated by the AGE-BSA treatment (Figure 4(c)). In addition, we examined the Sirt 1 activity in HUVECs with various treatments; and it was indicated in Figure 4(d) that the Sirt 1 specific inhibitor, EX527 (0.5 *μ*M), markedly downregulated the Sirt 1 activity in HUVECs, compared to in the control cells (Figure 4(d) columns 1 and 2; *P* < 0.001). And the Sirt 1 activity in HUVECs treated with 100 or 300 *μ*g/mL AGE-BSA also significantly decreased, dose dependently (Figure 4(d); *P* < 0.05 for 100 *μ*g/mL or *P* < 0.01 for 300 *μ*g/mL; *P* < 0.05 for 100 versus 300 *μ*g/mL). Thus, we confirmed in this section that the Sirt 1 was downregulated by AGEs in human endothelial cells.

### 3.4. Sirt 1 Regulates the AGE-Induced EndMT in HUVECs via Regulating TGF-*β*

**Figure 5:**
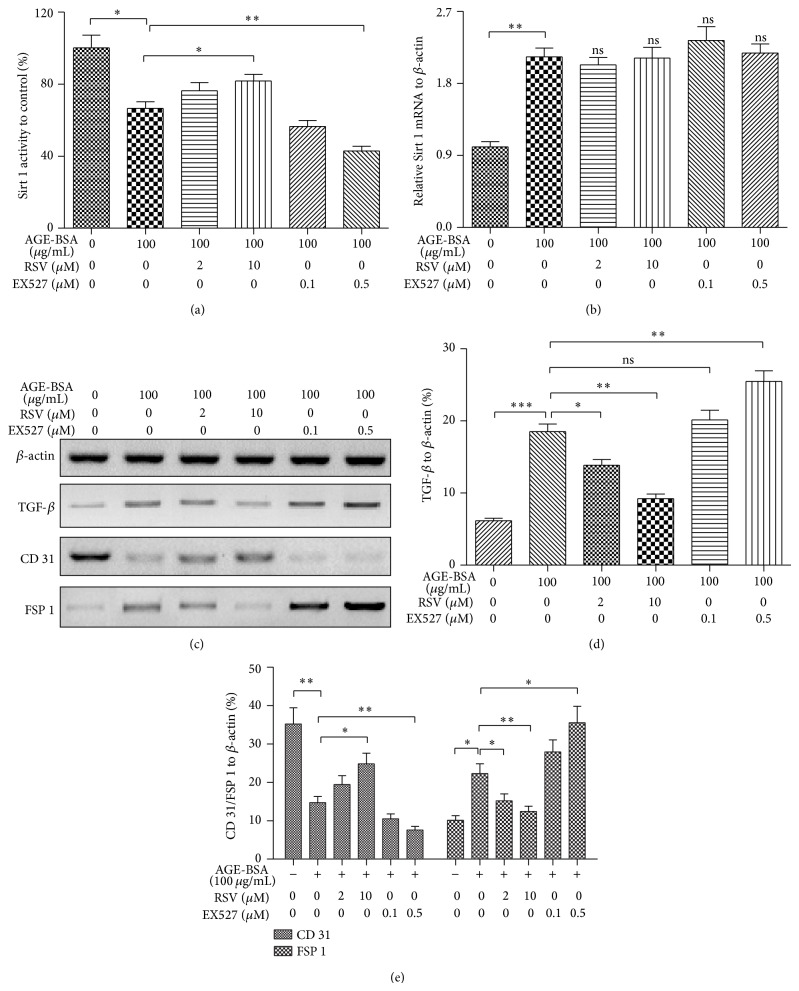
Chemical manipulation of Sirt 1 activity regulated the AGE-BSA-induced, TGF-*β*-mediated EndMT in HUVECs. (a) Regulation of Sirt 1 activity in the AGE-BSA-treated HUVECs by RSV (2 or 10 *μ*M) or by EX527 (0.1 or 0.5 *μ*M) for 48 h. (b) Sirt 1 mRNA level in HUVECs treated with 100 *μ*g/mL AGE-BSA and Sirt 1 regulators for 48 h. (c) Western blot analysis of TGF-*β*, CD 31, and FSP-1 in AGE-BSA-treated HUVECs, after Sirt 1 activity for 96 h. (d) Inhibition by RSV or aggravation by EX527 on TGF-*β* induction by AGE-BSA in HUVECs for 96 h. (e) Regulation by RSV or EX527 on the CD 31/FSP-1 in AGE-BSA-treated HUVECs. All results were averaged for three independent experiments. ^*^ *P* < 0.05, ^**^ *P* < 0.01, and ^***^ *P* < 0.001; ns: no significance.

To further recognize the regulatory role of Sirt 1 in the AGE-induced EndMT in HUVECs, we manipulated the Sirt 1 activity in the AGE-treated HUVECs with Sirt 1 activator and inhibitor [43]. The activator, resveratrol (RSV) with 10 *μ*M, ameliorated the Sirt 1 activity reduction (Figure 5(a), column 4 versus column 2; *P* < 0.05) which was induced by AGE-BSA treatment, whereas the Sirt 1 inhibitor, EX527 with 0.5 *μ*M, aggravated such Sirt 1 activity reduction significantly (Figure 5(a), column 6 versus column 2; *P* < 0.01). The Sirt 1 manipulation by RSV and EX527 also exerted regulation on the AGE-mediated Sirt 1 upregulation in mRNA level (Figure 5(b)). Moreover, the western blot assay demonstrated that the AGE-mediated TGF-*β* upregulation in protein level was influenced by the additional RSV or EX527 and RSV significantly inhibited the AGE-promoted TGF-*β*, whereas the EX527 aggravated such promotion significantly (Figures 5(c) and 5(d)) (*P* < 0.05 for 2 *μ*M RSV, *P* < 0.01 for 10 *μ*M RSV, and *P* < 0.01 for 0.5 *μ*M EX527).

We also analyzed the regulation, by Sirt 1 activator or inhibitor, on the AGE-induced CD 31 and FSP-1, which are, respectively, the markers of endothelial and mesenchymal cells, by western blot assay. And interestingly, both markers were regulated by RSV or EX527. Figures 5(c) and 5(e) indicated that the AGE-reduced CD 31 was inhibited by the RSV-mediated Sirt 1 activation significantly (column 4 versus column 2 in the “CD 31” group; *P* < 0.05), whereas the EX527-mediated Sirt 1 inhibition deteriorated the CD 31 reduction (column 6 versus column 2 in the “CD 31” group; *P* < 0.01). On the other side, the AGE-promoted FSP-1 was regulated by the two agents conversely; AGE-promoted FSP-1 was significantly blocked by either 2 or 10 *μ*M RSV (*P* < 0.05 or *P* < 0.01), whereas it was significantly aggravated by 0.5 *μ*M EX527. Thus, Sirt 1 was confirmed to regulate the AGE-induced EndMT TGF-*β* dependently.

In addition, to elucidate whether the Sirt 1 regulation on the EndMT was TGF-*β*-dependent, we examined the regulation by TGF-*β* on the Sirt 1 expression and Sirt 1 activity in HUVECs. It was shown in Supplemental Figure 1, in Supplementary Material available online at http://dx.doi.org/10.1155/2014/684242, that neither the mRNA nor the protein expression of Sirt 1 was regulated by TGF-*β* treatment with various doses and with various treating hours. And the Sirt 1 activity was also not regulated by the TGF-*β* treatment. Therefore, the Sirt 1 reduction in the AGE-induced EndMT was TGF-*β*-independent.

## 4. Discussion

It has been established that high levels of blood glucose could lead to the development of complications in DM via posing oxidative stress, protein kinase C activation, and the accumulation of AGEs [10–13]. The* in vivo* accumulation of AGEs over time has been confirmed to cause structural and functional changes of vascularity [53], such as arterial stiffening, atherosclerotic plaque formation, and endothelial dysfunction, which resulted from either non-receptor- or receptor-mediated mechanisms. For example, the non-receptor-mediated crosslinking of collagen with AGE produces stiffness of blood vessels [54], and the RAGE-AGE interaction activates oxidative stress in endothelial cells [55]. AGEs have also been shown to induce EMT or EndMT through RAGE-ERK1/2 MAP kinase signaling pathway [56], via protein kinase B signaling cascades [57] or by the mediation of connective tissue growth factor [23]. Recently, AGEs have been shown to induce the expression of fibronectin and TGF-*β* by activating glomerular mesangial cells, and the induction can be inhibited by Sirt 1 [58], implying a possible role of TGF-*β* and Sirt 1 in the AGE-induced EMT or EndMT.

In the present study, we confirmed the regulation by AGEs in HUVECs on the CD 31 and such mesenchymal markers as FSP-1, *α*-SMA, and collagen I. AGE-BSAs significantly reduced the CD 31 expression in both mRNA and protein levels in HUVECs, compared to the BSA, with a dose dependence. On the other side, FSP-1, *α*-SMA, and collagen I were significantly upregulated by the AGE-BSAs treatment, rather than the BSA treatment, dose dependently. EndMT shares lots of signaling pathways with EMT, such as the TGF-*β*1 signaling [47, 48]. We then examined the TGF-*β*1 activation in the HUVECs after AGE treatment. Results demonstrated a significant promotion of AGEs not only to RAGEs [49, 50], but also to the TGF-*β*1 and TGFR I markedly with a dose dependence. Taken together, the AGE-BSA promoted the activity of TGF-*β* in the human endothelial cells. Recently, Sirt 1 has been reported to decrease in diabetes [51, 52] and is confirmed to resist AGE-induced EMT in glomerular mesangial cells [43]. To identify whether there is a feedback loop in the Sirt 1-AGE regulation signaling, we investigated the regulation on Sirt 1 by AGE in endothelial cells, and it was shown that the Sirt 1, rather than Sirt 2, was significantly downregulated by the AGE-BSA in both mRNA and protein levels dose dependently. In addition, the Sirt 1 activity in HUVECs treated with AGE-BSA was also significantly reduced dose dependently. Thus, we confirmed that the Sirt 1 was downregulated by AGEs in human endothelial cells.

The sirtuin family is widely distributed from eubacteria to eukaryotes, including humans, in which there are seven different homologous proteins, with a central highly conserved region, defined as the catalytic core [59]. Sirt 1 is a NAD+-dependent deacetylase closely related to yeast Sir 2, the first gene discovered in sirtuin family [60]. Dependence of Sirt 1 on NAD+ levels infers that it acts as a key sensor for changes in metabolism. Sirt 1 has a range of targets involved in regulating the metabolism at various tissues. In particular, the regulatory role of Sirt 1 has been recently uncovered in the hepatic glucose homeostasis [61–63], in pancreatic *β* cells insulin secretion [64, 65], in skeletal muscle metabolic homeostasis [66, 67], and in adipocyte energy homeostasis and insulin sensitization [68]. And Sirt1 has been shown to inhibit the AGEs-induced expressions of fibronectin and TGF-*β*1 in glomerular mesangial cells [43]. Thus, the downregulated Sirt 1 might be also involved in the regulation of AGE-induced EndMT in HUVECs.

We then examined the regulatory role of Sirt 1 in the AGE-induced EndMT in HUVECs by manipulating the Sirt 1 activity in the AGE-treated HUVECs with Sirt 1 activator and inhibitor [43]. The activator, RSV treatment, ameliorated the AGE-BSA-mediated Sirt 1 activity reduction, whereas the Sirt 1 inhibitor, EX527, aggravated such Sirt 1 activity reduction significantly. Moreover, both RSV and EX527 markedly inhibited or aggravated the AGE-mediated TGF-*β* upregulation. We also analyzed the regulation, by RSV or EX527, on the AGE-induced CD 31 and FSP-1, which are, respectively, the markers of endothelial and mesenchymal cells. And interestingly, both markers were regulated by RSV or EX527: the AGE-reduced CD 31 was inhibited by the RSV-mediated Sirt 1 activation, whereas it was deteriorated by the EX527-mediated Sirt 1 inhibition. On the other side, the AGE-promoted FSP-1 was regulated by the two agents conversely; AGE-promoted FSP-1 was significantly blocked by RSV, whereas it was significantly aggravated by EX527. Thus, Sirt 1 was confirmed to regulate the AGE-induced EndMT via TGF-*β*. Additionally, we examined the regulation by TGF-*β* on the Sirt 1 expression and Sirt 1 activity in HUVECs, and neither expression of Sirt 1 in both mRNA and protein levels nor the Sirt 1 activity was regulated by TGF-*β* treatment with various doses and with various treating hours. Therefore, the Sirt 1 reduction in the AGE-induced EndMT was TGF-*β*-independent.

In summary, we found in the present study that AGE-BSA induced EndMT, as indicated by downregulated CD 31 and upregulated FSP-1, *α*-SMA, and collagen I, in HUVEC via upregulating TGF-*β* and downregulating Sirt 1, which also negatively regulated TGF-*β* in the cell. This study indicated the direct injury by AGE-BSA in endothelial cells and implied the EndMT probably as an important mechanism of AGE-induced cardiovascular injury.

## Supplementary Material

Supplemental Figure 1. Regulation on Sirt 1 expression by TGF-*β*.
