# Supplementary material for: Advanced Glycation End Products Induce Endothelial-to-Mesenchymal Transition via Downregulating Sirt 1 and Upregulating TGF-β in Human Endothelial Cells
Source: Biomed Res Int. 2015 Feb 1;2015:684242. doi: 10.1155/2015/684242 (PMC4330956; doi:10.1155/2015/684242)
Supplement: Supplementary file 1 — Supplemental Figure 1. Regulation on Sirt 1 expression by TGF-β. [file 684242.f1.pdf]

Supplemental Figure 1. Regulation on Sirt 1 expression by TGF-β

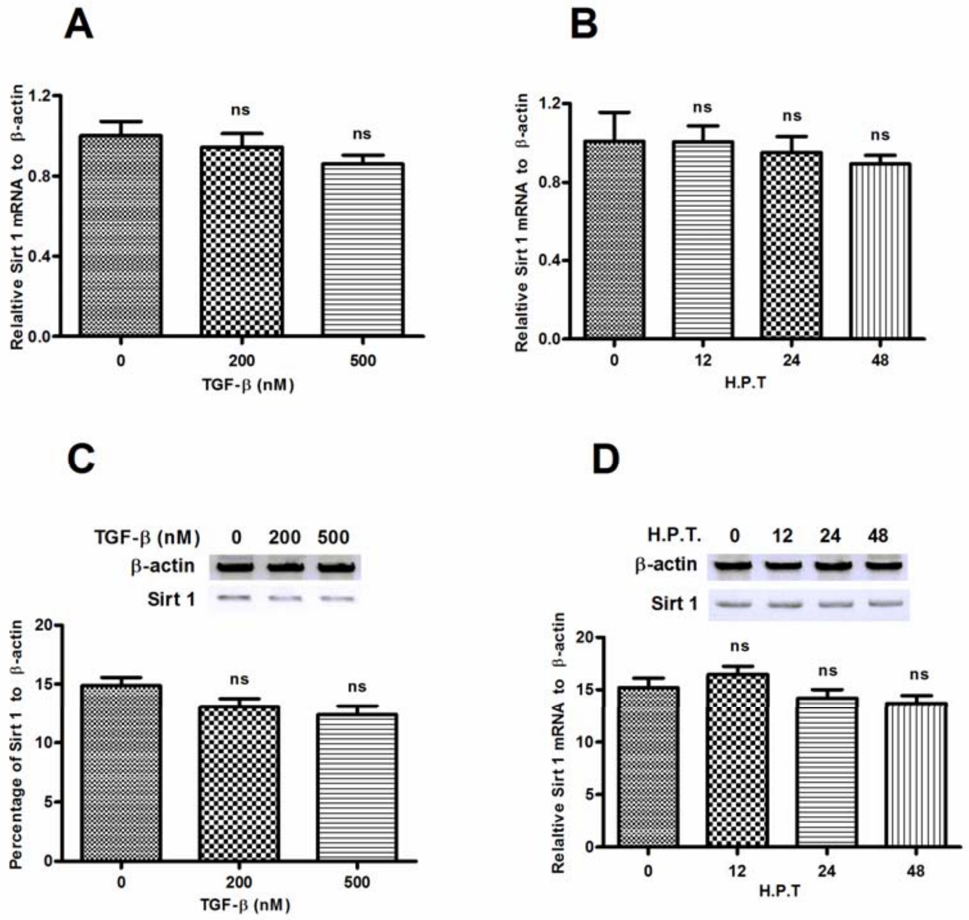

A and B: Regulation on Sirt 1 expression in mRNA level by TGF-β treatment with various dose (A) OR with 500 nM for the inoculation for various hours (B); C and D: Regulation on Sirt 1 expression protein level by TGF-β treatment with various dose (C) OR with 500 nM for the inoculation for various hours (D).
